# Osteoporosis

**DOI:** 10.1093/emph/eov032

**Published:** 2015-12-29

**Authors:** Ian J. Wallace, Clinton T. Rubin, Daniel E. Lieberman

**Affiliations:** ^1^Department of Human Evolutionary Biology, Harvard University, Cambridge, Massachusetts, USA and; ^2^Department of Biomedical Engineering, Stony Brook University, Stony Brook, New York, USA

## OSTEOPOROSIS

Osteoporosis is a disease of compromised bone strength increasing susceptibility to fracture. The standard clinical diagnostic criterion is a bone mineral density score 2.5 SD or more below the population average for young adults. Bone densities overlap, however, among people who do and do not experience fractures, indicating that factors other than bone mass affect fracture risk. Tissue architecture, in particular, is also critical to skeletal mechanical integrity.

Before adulthood, bone metabolism is biased toward net anabolism, with the greatest tissue gains occurring on the periosteal surface. By approximately age 30, bone mass peaks; and thereafter, bone turnover shifts toward catabolism, with losses occurring mainly on the endosteal surface and in trabecular bone. Rates of bone loss can be accelerated by age-related shifts in hormone levels, particularly the post-menopausal drop in estrogen. In sum, osteoporosis results from suboptimal bone mass and architecture achieved during growth plus considerable adult bone loss. Fracture risk is further exacerbated by muscle wasting and poor balance, which increase the tendency to fall and reduce the ability to protect one’s self during a fall.

Although susceptibility to osteoporosis is influenced by intrinsic factors such as age, sex and genetics, it is generally a preventable disease. Essential for the prevention of osteoporotic fractures are healthy lifestyle habits, especially routine physical activity and sufficient intake of vitamin D and calcium, which help maximize peak bone mass and skeletal rigidity, retard bone loss and improve muscle strength and balance [1, 2].

## EVOLUTIONARY PERSPECTIVES

Prior to the industrial era, osteoporotic fractures were exceedingly rare in human evolutionary history, even among the elderly [3]. Moreover, not all contemporary populations around the world are at similar risk. Age-standardized rates of osteoporosis-related hip fractures differ by at least 10-fold between countries exhibiting the highest and lowest incidence, with people in highly industrialized nations being at greatest risk [4]. Implicitly, these evolutionary and global trends indicate that osteoporosis fits the criteria of a ‘mismatch disease’ caused by our skeletons being inadequately or imperfectly adapted to novel conditions associated with industrialized environments, especially physical inactivity, as well as diets lacking vital nutrients [5, 6]. The etiology of osteoporosis thus parallels that of the obesity pandemic and other by-products of activity- and diet-induced metabolic imbalances that plague industrialized societies [6].

Of the novel environmental factors that increase osteoporosis risk, low physical activity levels among children and adolescents in many industrialized countries are especially problematic, since it is during these years that bones develop their maximum strength. While physical activity before skeletal maturity increases the rate of periosteal bone growth, thereby enhancing bone thickness and structural resistance to bending, activity during adulthood mainly slows bone loss [7]. Thus, there is a window of opportunity for physical activity to augment bone strength; and this window is narrowing in industrialized nations as puberty occurs at progressively earlier ages [8]. Declining commitment to physical education in schools is worsening the situation.

## FUTURE IMPLICATIONS

Osteoporosis prevalence is increasing as more physically inactive people with low bone quantity and quality become elderly. For these individuals, pharmaceutical options exist that can delay age-related bone loss, but all have side effects and contraindications, and can only be used for a limited time. Moreover, these drugs target only bone, whereas fracture risk reflects deterioration of the entire musculoskeletal system. Evolutionary evidence that osteoporosis is not an inevitable outcome of aging highlights that the best strategy for countering the disease is prevention through routine physical activity and beneficial nutrition throughout life, but especially prior to maturity, so that people enter old age with more osteoporosis-resilient skeletons.

## References

[1] Kannus P Uusi-Rasi K Palvanen M Non-pharmacological means to prevent fractures among older adults. Ann Med 2005; 37:303–10. 16019730 10.1080/07853890510007197

[2] Rubin CT Rubin J Judex S Exercise and the prevention of osteoporosis In: Rosen CJ (ed.). Primer on the Metabolic Bone Diseases and Disorders of Mineral Metabolism. Hoboken: Wiley, 2013, 396–402.

[3] Agarwal SC Grynpas MD. Bone quantity and quality in past populations. Anat Rec 1996; 246:423–32. 8955781 10.1002/(SICI)1097-0185(199612)246:4<423::AID-AR1>3.0.CO;2-W

[4] Cauley JA Chalhoub D Kassem AM Geographic and ethnic disparities in osteoporotic fractures. Nat Rev Endocrinol, 2014; 10:338–51. 24751883 10.1038/nrendo.2014.51

[5] Karasik D. Osteoporosis: an evolutionary perspective. Hum Genet 2008; 124:349–56. 18781328 10.1007/s00439-008-0559-8

[6] Lieberman DE. The Story of the Human Body. New York: Pantheon; 2013.

[7] Pearson OM Lieberman DE. The aging of Wolff’s “Law”: ontogeny and responses to mechanical loading in cortical bone. Yearb Phys Anthropol 2004; 47:63–99. 10.1002/ajpa.20155 15605390

[8] Devlin MJ. Estrogen, exercise, and the skeleton. Evol Anthropol 2011; 20:54–61 22034104 10.1002/evan.20299

